# Synergistic Photoelectrochemical and Photocatalytic Properties of the Cobalt Nanoparticles-Embedded TiVO_4_ Thin Film

**DOI:** 10.1021/acsomega.3c02089

**Published:** 2023-07-24

**Authors:** Manal Alruwaili, Anurag Roy, Mansour Alhabradi, Xiuru Yang, Asif Ali Tahir

**Affiliations:** † Solar Energy Research Group, Environment and Sustainability Institute, Faculty of Environment, Science and Economy, University of Exeter, Penryn TR10 9FE, U.K.; ‡ Physics Department, Faculty of Science, Jouf University, P.O. Box 2014, Sakaka 42421, Saudi Arabia; § Department of Physics, Faculty of Science, Majmaah University, Majmaah 11952, Saudi Arabia

## Abstract

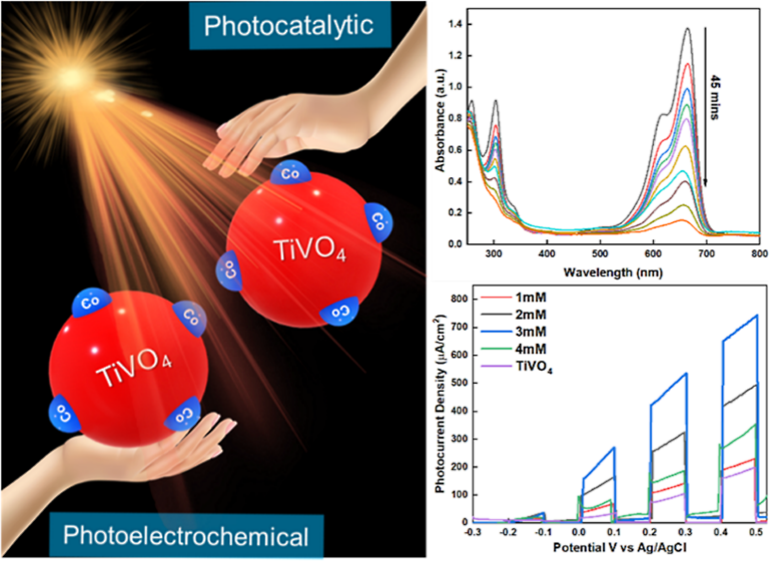

To optimize the semiconductor properties of TiVO_4_ thin films and enhance their performance, we incorporated cobalt nanoparticles as an effective co-catalyst consisting of a non-noble metal. Through an investigation into the impact of cobalt loading on spray pyrolyzed TiVO_4_ thin films, we observed a significant enhancement in the photoelectrochemical (PEC) performance. This was accomplished by carefully optimizing the concentrations of Co^2+^ (3 mM) to fabricate a composite electrode, resulting in a higher photocurrent density for the TiVO_4_:Co photoanode. When an applied potential of 1.23 V (vs RHE) was used, the photocurrent density reached 450 μA/cm^2^, approximately 5 times higher than that of bare TiVO_4_. We conducted a thorough characterization of the composite structure and optical properties. Additionally, electrochemical impedance spectroscopy analysis indicated that the TiVO_4_/Co thin film exhibited a smaller semicircle, indicating a significant improvement in charge transfer at the interface. In comparison to bare TiVO_4_, the TiVO_4_/Co composite exhibited a notable improvement in photocatalytic activity when degrading methylene blue (MB) dye, a widely employed model dye. Under light illumination, a TiVO_4_/Co thin film exhibited a notable dye degradation rate of 97% within a 45 min duration. The scalability of our fabrication method makes it suitable for large-area devices intended for sunlight-driven PEC seawater splitting studies.

## Introduction

Photocatalytic and photoelectrochemical (PEC) systems used for solar-to-chemical energy conversion applications are developed in parallel. While both systems employ photoactive semiconductors as the core component in their functionality, some common strategies have been formulated and adopted to improve their performance. PEC water splitting has attracted more attention recently as one of the most promising H_2_ production methods due to its utilization of the unlimited energy source of solar light without producing any carbon dioxide emissions and environmental pollutants. Photovoltaic (PV) solar cell technology directly converts solar energy into electricity. The PEC water splitting process consists of three steps: light absorption resulting in charge carrier generation, transportation of charge to the surfaces, and the utilization of excited photocarriers to drive overall required reactions. Therefore, transporting the photo-generated carriers from a photo-absorber to a solid/liquid interface where catalytic sites can oxidize or reduce the water is critical.^1−4^ Fujishima and Honda were the first observers of the phenomenon of water splitting, using n-type TiO_2_ as a photoanode since early 1972.^5^ Subsequently, research has been devoted to achieving high performance of PEC systems, taking into account the fundamental considerations such as potential requirement, diffusion length, bulk defects, and charges recombinations.^6^

Metal oxide semiconductors such as Fe_2_O_3_,^7^ Co_3_O_4_,^8^ TiO_2_,^9^ BiVO_4_,^10^ Fe_*x*_V_*x*_O_4_,^11^ and Cu_*x*_V_*x*_O_*x*_^12^ have been used intensively in the photocatalytic applications, showing some effective photoactive performances under solar irradiation. However, several drawbacks are more likely to respond to their low efficiency, e.g., band gap structures that cannot straddle some redox potentials to drive overall water splitting reactions. Consequently, charge carriers’ recombination rate is swift, and the slow transport and trapping of charge carriers occur on the surface.^7−9,13^ So, numerous strategies such as doping with other metals,^14,15^ forming a heterostructure,^16,17^ and co-catalyst deposition^18,19^ have been explored to overcome poor metal oxide electrode PEC performance.

The main keys to using the co-catalyst approach as impurity implantation on the photocatalyst’s surface for PEC water splitting are to extend these materials’ spectral response toward visible light by enhancing the light harvesting, facilitating charge transfer, and providing active sites of these materials.^20^ Additionally, co-catalysts can lower the overpotential of the hydrogen evolution (HER) and oxygen evolution (OER) reactions, as sequence provides long-term stability.^21,22^ Nobel (Pt, Au, Ag, and Pd) and non-noble (Co, Ni, and Cu) metals have been explored as the metal ion co-catalyst with another metal oxide for photocatalysis applications, showing excellent H_2_ activity with an improvement in the photocurrent density compared with that of those un-coupling materials with co-catalysts.^19,23−27^

Ag, Au, and Ni are advantageous nanoparticles (NPs) loaded on the semiconductor’s surfaces owing to the surface plasmonic resonance (SPR) effect, which plays as mediators of electrons at the solid/liquid interface. Pawar et al. (2018) evaluated the SPR effect of Ag and Ni NPs loading on LaFeO_3_ thin film surfaces. Ag, Ni/LaFeO_3_ exhibited higher light absorption, resulting in an increase in the current density up to 0.074 and 0.066 mA/cm^2^ at 0.6 V vs RHE compared to that of bare LaFeO_3_, respectively.^18,19^ In their study, Reichert et al. (2015) investigated the role of Au/TiO_2_ electrode thin films in the production of O_2_ and H_2_ at the same time via varying Au NP loading on the produced films. The researchers observed that the intensity of light varied depending on the quantity of Au NPs, and these reactions acted as catalysts, leading to an enhanced separation of photo-generated charge carriers.^28^ Meanwhile, Siavash Moakhar et al. studied the effect of using dual co-catalysts of AuPd bimetallic NPs deposited on the TiO_2_ nanorod arrays via the electrodeposition technique. AuPd–TiO_2_’s structure depicted an enhanced photocurrent density of 3.36 mA/cm^2^ under AM 1.5 light illumination (100 mW cm^–2^) due to hindering charge carrier recombination and passivating surface defects shown on the TiO_2_ nanorods. As a result, the AuPd– TiO_2_ film exhibited combined properties from both catalysts: Au by increasing light harvesting capacity and Pd properties by accelerating electrocatalytic activity.^29^ Moreover, utilization of the NP co-catalyst onto photocatalysts has also shown excellent performance in the photocatalytic dye degradation and removal of the environmental pollutants applications.^30,31^ The presence of co-catalyst NPs can provide the additional active site with large contact surface area, enhancing the reactions and resulting in high efficiency with superior photocatalytic activity.^32^

Given the exorbitant expense and limited availability of noble metals, the imperative of developing affordable and exceptionally efficient cocatalysts has become apparent in facilitating the widespread implementation of photocatalytic water splitting. Moreover, it has a reduced tendency to form recombination centers, leading to enhanced and efficient water splitting reactions, specifically the OER and HER. Consequently, incorporating this co-catalyst results in a significant improvement in the PEC activity.^33−35^ Co NPs, an earth-abundant transition co-catalyst, were introduced onto the surface of plain TiVO_4_ thin films using the wet impregnation technique inspired by our previous work.^36^ In this study, we aimed to enhance the photocurrent density of the TiVO_4_ photoanode, which was previously prepared via spray pyrolysis, by varying the cobalt solution contents. The presence of an optimal Co loading of 3 mM resulted in a significantly improved PEC performance and excellent photocatalytic dye degradation compared to that of bare TiVO_4_. These prepared films were employed as photocatalysts to remove methylene blue (MB) dye from wastewater. The thin films exhibited superior charge carrier separation compared to their powder form counterparts and demonstrated relatively faster separation from an aqueous solution during photocatalytic degradation.

## Materials and Methods

### Materials

To fabricate a TiVO_4_/Co thin film, chemicals such as vanadium acetylacetonate, titanium isopropoxide, ethanol, and trifluoroacetate acid were procured from Merck Life Science Products (U.K) and employed without additional purification. The photodegradation efficiency of the optimized TiVO_4_/Co (3 mM) thin film was evaluated using Merck Life Sciences’ MB dye.

### Fabrication of the TiVO_4_ Photoanode

Titanium vanadate photoanodes were synthesized using the spray pyrolysis technique, as detailed in our previous publication.^36^ In summary, a solution was prepared by dissolving vanadium acetylacetonate and titanium isopropoxide in 15 mL of ethanol. Subsequently, 0.05 mL of trifluoroacetate acid (99%) was added to the solution and stirred for 2 h. The solution was sprayed onto cleaned fluorine-doped tin oxide (FTO) glasses measuring 1 cm × 1 cm while maintaining a substrate temperature of 250 °C. The coated substrates were annealed at 600 °C for 2 h in a muffle furnace and then cooled to room temperature.

### Fabrication of the Co-incorporated TiVO_4_ (TiVO_4_/Co) Photoanode

The FTO substrates coated with TiVO_4_ were placed in a solution containing 0.7 mM sodium citrate, along with varying concentrations of cobalt nitrate (1, 2, 3, and 4 mM).^8^ The immersion took place at a temperature of 100 °C for a duration of 2 h. Subsequently, the TiVO_4_/Co films were washed with deionized water and left to dry in air. These prepared films were then utilized for subsequent PEC performance testing.

### Photocatalytic Dye Degradation Test

For the experiment, 10 mg of pure MB powder was dissolved in 1 L of deionized water to achieve a concentration of 10 mg/L. The prepared film was immersed in 30 mL of the MB solution, placed in a cylindrical Pyrex container, and subjected to constant stirring in the dark for 30 min to establish adsorption/desorption equilibrium. Subsequently, to simulate 1 sun condition with an approximate light intensity of 100 mW/cm^2^, a Newport 66902, 300 W xenon lamp with an air mass (AM) of 1.5 was employed. Following the designated test period, the UV–vis spectrophotometer was used to measure the absorbance of the solution, which indicated the degradation of MB.

### Materials Characterization

To understand the structure and phases of the TiVO_4_–Co thin films, a Bruker D8 X-ray diffractometer was utilized in conjunction with monochromatic Cu kα (λ = 0.154 nm) radiation. Morphological thin film analysis was conducted using the TESCAN VEGA3 scanning electron microscope with an energy-dispersive spectroscopy (EDS) system provided by Oxford Instruments. Additionally, the structural characterization involved high-resolution transmission electron microscopy (HR-TEM), selected area electron diffraction (SAED), and scanning transmission electron microscopy (STEM) performed using the JEOL JEM-2100F transmission electron microscope operating at 200 kV. X-ray photoelectron spectroscopy (XPS) analysis was carried out using a Thermo NEXSA XPS instrument equipped with a monochromated Al kα X-ray source (1486.7 eV). The thin film data were acquired under a pressure below 10^–8^ Torr and at a room temperature of 294 K. CasaXPS v2.3.20PR1.0 software was employed for data analysis, and calibration was performed using the C 1s peak at 284.8 eV. The absorption spectra of the thin films were obtained using Perkin Elmer’s UV-vis-NIR UV-3600 Plus spectrophotometer.

The TiVO_4_/Co photoanode was utilized for PEC studies using the Metrohm Autolab (PGSTAT302N) workstation with three-electrode compartments. The reference electrode was a saturated aqueous solution of Ag/AgCl in KCl, while the electrolyte for the electrochemical testing was a 1 M aqueous solution of NaOH with a pH of 13.6. To simulate 1 SUN condition (100 mW/cm2), light intensity was generated using a Newport setup consisting of a 300 W xenon lamp with an AM 1.5 filter and a 420 nm cut-off filter to eliminate ultraviolet radiation. The voltage of the photoanode (measured against Ag/AgCl) was recorded at a scan rate of 0.01 V/s, ranging from negative to positive potentials (from −0.3 V to +0.5 V) under light, dark, and chopping conditions. Subsequently, all potentials were converted to a reversible hydrogen electrode (RHE) potential using the Nernst equation given in eq 1
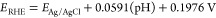
1where the pH of the electrolyte was kept at 13.6. Furthermore, electrochemical impedance spectroscopy (EIS) was performed in the frequency range of 10^–1^–10^5^ Hz in 1 M NaOH aqueous solution under 1 SUN illumination (100 mW/cm^2^). The Mott–Schottky equation was used to determine the photoanode’s flat band potential (*V*_fb_) and concentration of the dopants (*N*_D_) following the formula
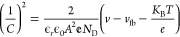
2where *C* is space charge capacitance, ε_0_ is the permittivity of vacuum, ε_r_ is the relative permittivity of a material, *A* is the area of the film, *N*_D_ is the carrier concentration, *K*_B_ is the Boltzmann constant, *T* is the temperature, *e* is the electronic charge, *V* is the applied potential, and *V*_fb_ is the flat band potential, which is estimated through a linear fit in the Mott–Schottky plot.

For photocatalytic dye degradation, a UV–vis spectrophotometer was used to reflect the degradation of the MB dye. The degradation of the MB dye was performed using an ozone-free light source of 300 W from Newport Model: 66483-300XF-R22.

The rate of degradation was calculated using the following eq 2
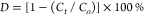
3where *C*_ο_ is the initial concentration of the dye solution and *C*_t_ is the remaining concentration after irradiation at the tested time.

## Results and Discussion

### Characterization of the Photocatalyst Thin Film

#### Optical Analysis

**Figure 1:**
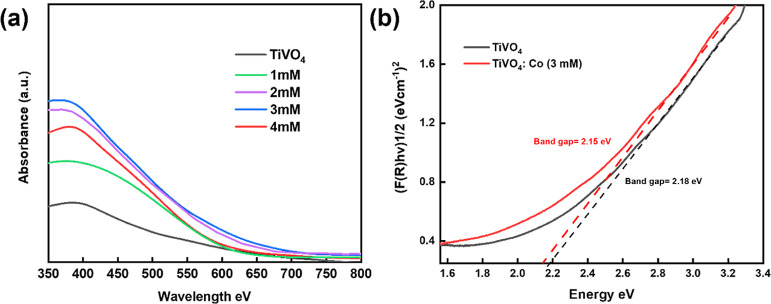
(a) Absorbance spectra of various TiVO_4_/Co films and (b) Kubelka–Munk plots of bare and TiVO_4_/Co (3 mM) thin films.

Figure 1a displays the UV–vis absorption spectra of various concentrations of Co loaded on bare TiVO_4_. Higher absorption is observed in the shorter wavelength range (400–550 nm). Furthermore, increased light absorbance beyond 550 nm is attributed to Co loading on the bare film, although it should be relatively small since more incident light is absorbed with higher Co loading.^37^ Additionally, it is noted that the absorption edges remain almost unchanged after loading Co particles, indicating Co deposition on the surface rather than doping into the TiVO_4_ lattice.^38^ The band gap of the optimally loaded Co NPs is calculated using the Kubelka–Munk function from reflection spectra, resulting in an estimated value of 2.15 eV, slightly decreased by 0.03 eV compared to that of the bare film (Figure 1b). Conversely, excessive Co loading (4 mM) reduces light absorption due to scattering from agglomerated Co on the surface, obstructing more active sites of the photocatalyst.^39^ These findings provide evidence that the amount of co-catalyst loading significantly affects photocatalytic absorption as a higher co-catalyst concentration can shield incident light, leading to decreased photocatalytic activity. Thus, considering the effect of co-catalyst loading on absorption spectra, a concentration of 3 mM is deemed optimal for Co loading as it exhibits maximum absorption, warranting further analysis of PEC and photocatalytic degradation performance.

**Figure 2:**
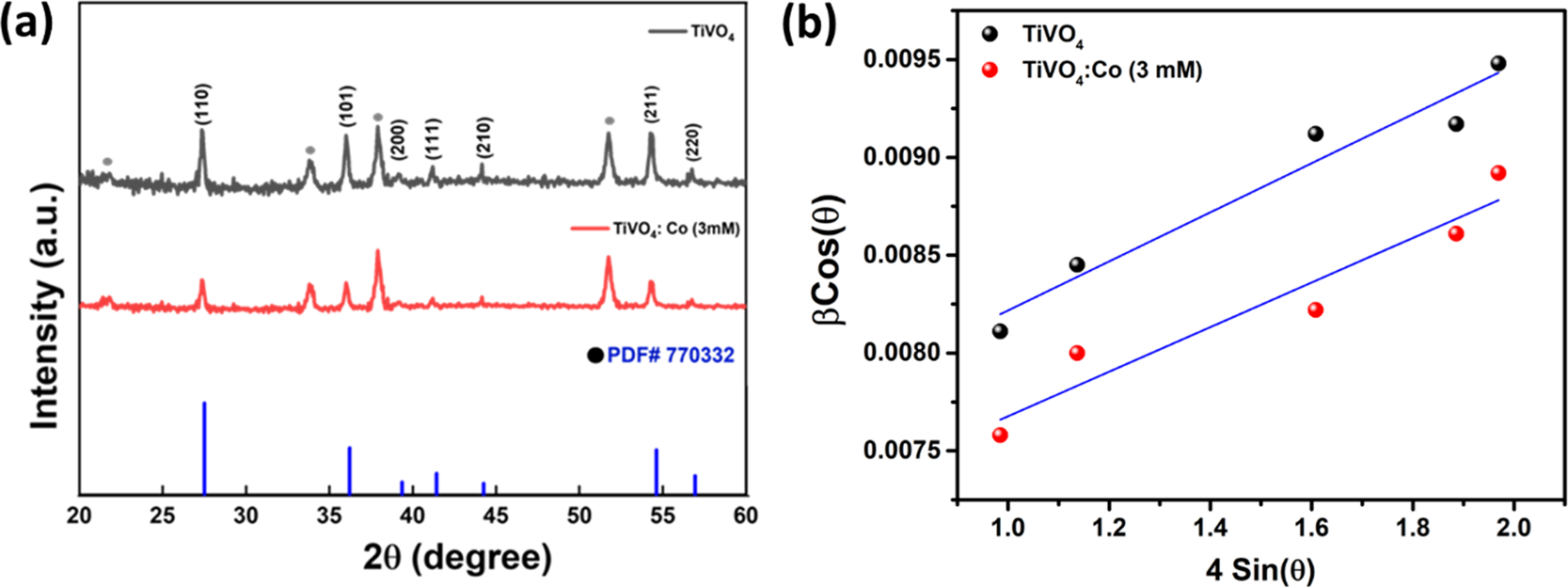
(a) XRD patterns of bare TiVO_4_ and TiVO_4_/Co photoanodes deposited on FTO glass and (b) corresponding Williamson–Hall analysis plot.

The X-ray diffraction (XRD) patterns of the prepared TiVO_4_ and TiVO_4_/Co (3 mM) samples are displayed in Figure 2a. Both patterns exhibit diffraction peaks that match the single-phase TiVO_4_ peaks, specifically oriented in the (110), (111), and (211) crystal planes, which align well with previous findings.^36^ These results further confirm the tetragonal structure of the samples, as indicated by the JCPDS file 01-770332. The presence of additional peaks (depicted as gray circles) in the observed spectrum can be attributed to the FTO glass substrate. However, it is noteworthy that the distinct peaks associated with cobalt are not present in the TiVO_4_/Co (3 mM) film. This absence could be explained by either the small size of the Co particles or the low amount of Co loaded onto bare TiVO_4_, as previously mentioned.^26,40,41^ However, despite the absence of Co peaks, the intensity of the film peaks decreased upon Co loading without any noticeable shift. XRD patterns are influenced not only by the size of crystallites but possibly also by lattice strain and lattice defects. Moreover, the Williamson–Hall analysis is an effective method for distinguishing deformation peaks caused by variations in both the size and strain of the armature. The obtained lattice strain value from the Williamson–Hall plot (Figure 2b) for TiVO_4_/Co is 1.25 × 10^–3^, slightly higher than that of the pure TiVO_4_ sample (1.14 × 10^–3^).^42^ This indicates that the inclusion of cobalt led to a minor strain in the crystallite size, demonstrating the successful deposition of Co particles onto the TiVO_4_ lattice without causing distortion. Furthermore, there is the negligible disparity in the crystallite sizes between bare TiVO_4_ (21.3 nm) and TiVO_4_/Co (19.9 nm), suggesting that Co^2+^ has not been doped into the TiVO_4_ lattice. However, considering the strain aspect, it is highly likely that Co^2+^ may have been deposited either as Co NPs or surface-adsorbed onto the TiVO_4_ crystallite during the incorporation process.^43^

#### Microstructural Analysis

**Figure 3:**
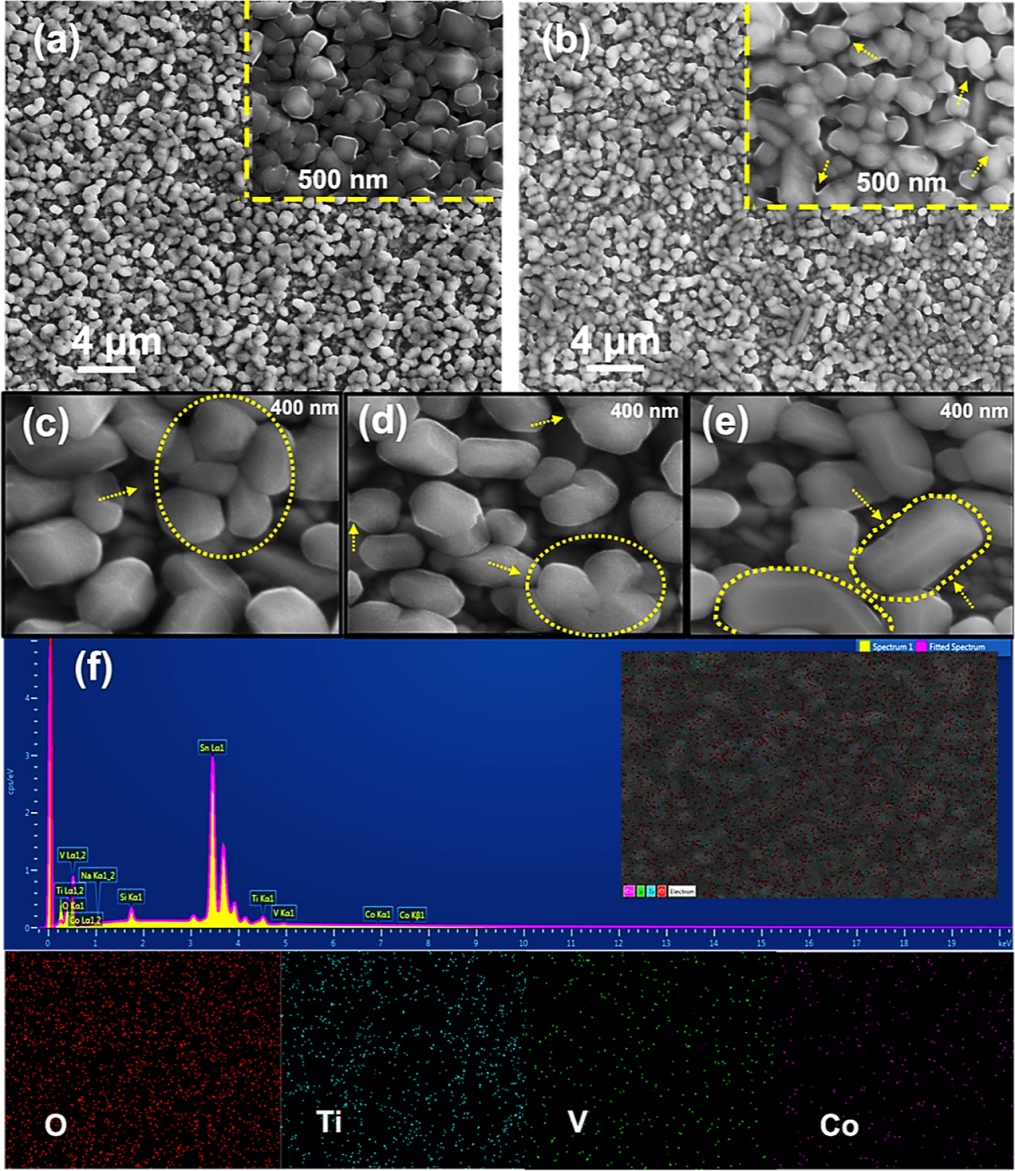
SEM microstructural images of (a) bare TiVO_4_, (b) TiVO_4_/Co (3 mM), (c) TiVO_4_/Co (1 mM), (d) TiVO_4_/Co (2 mM), and (e) TiVO_4_/Co (4 mM) photoanodes and (f) EDS spectrum of the TiVO_4_/Co (3 mM) photoanode.

Figure 3a–e presents top-view scanning electron microscopy (SEM) images of different films: bare TiVO_4_ and TiVO_4_ loaded with Co at various solution contents. In Figure 3a, the bare TiVO_4_ film exhibits a porous structure with well-sized grains and a smooth surface, consistent with previous findings. Upon loading Co (3 mM) onto the TiVO_4_ surface, which showed the highest photocurrent, the surface remains relatively smooth, with a uniform particle distribution. The spatial structure of TiVO_4_ remains intact after loading, with a more visible and rougher adhered-like structure on the tail side of TiVO_4_ particles, as shown in the inset of Figure 3b.

No significant changes in morphology are observed when a small amount of Co particles (1 mM and 2 mM) is loaded onto the bare TiVO_4_ surface, as seen in Figure 3c and 3d. However, these samples display increased porosity, the gathering of primary particles, and formation of an ultra-thin layer. Figure 3e reveals that an excessive amount of the Co catalyst results in non-uniform primary particle sizes, obscured edges covered by a thick layer, and the formation of larger, varying grain sizes due to particle agglomeration.

Compared to the structure of the bare film, the TiVO_4_/Co film demonstrates an appropriate structure for achieving a moderate porosity size and active contact sites with the electrolyte. This facilitates the separation and transfer of charges, thereby enhancing the photocurrent density. Consequently, the presence of Co particles on the TiVO_4_ surface improves the intrinsic photocatalytic activity of the compound.^21^Figure 3f presents the EDS analysis of TiVO_4_/Co, confirming the presence of V, Ti, O, and Co elements. The elemental surface scanning image shows a uniform distribution of Co particles on the TiVO_4_ surface.

**Figure 4:**
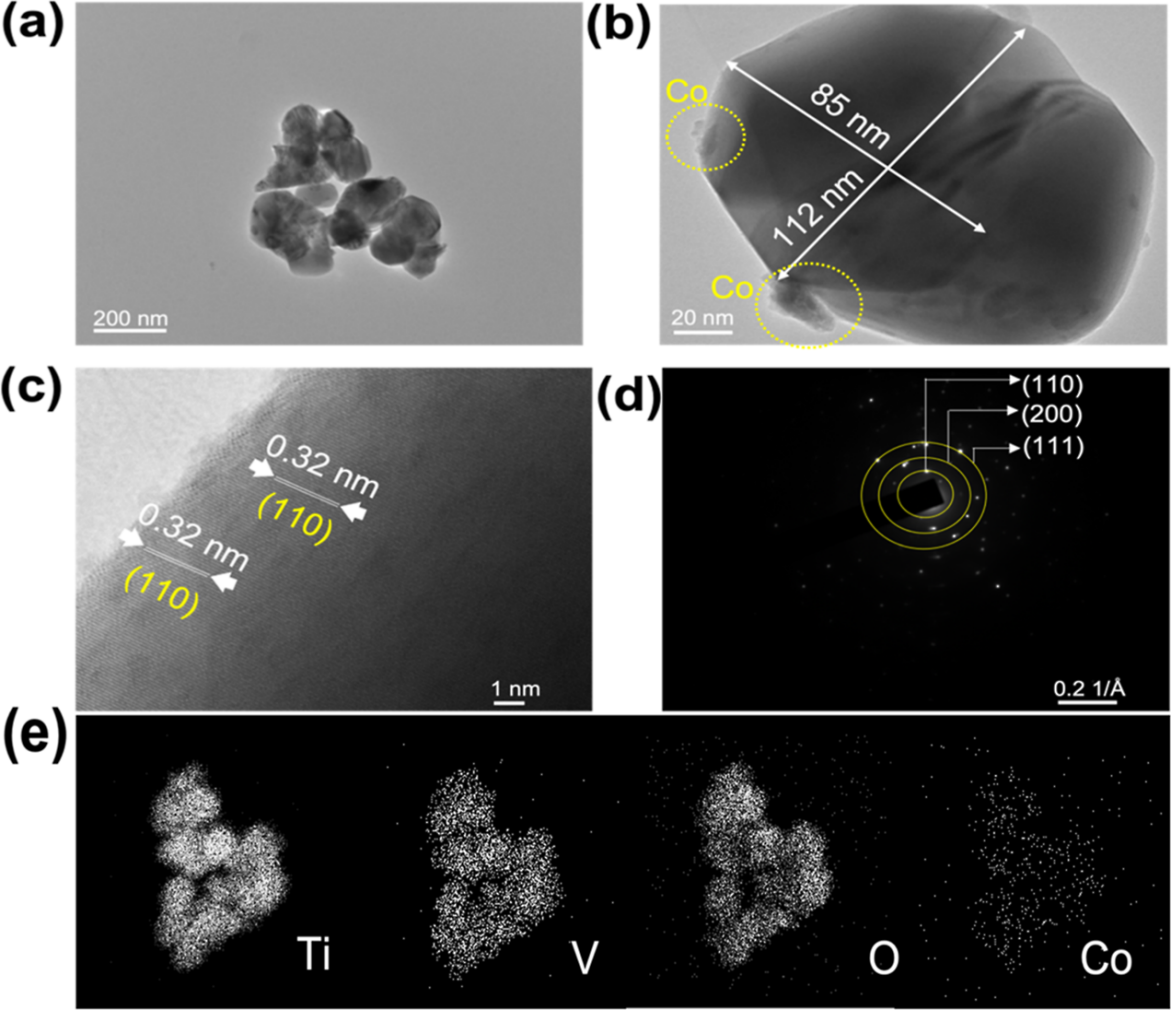
(a,b) TEM bright field images at different magnifications, (b) corresponding HRTEM images, and (d) SAED and (e) STEM images of the TiVO_4_/Co (3 mM) thin film.

Figure 4a,b illustrates the morphology of the Co-loaded TiVO_4_ thin film, consisting of spatially interconnected quasi-spherical TiVO_4_ NPs at their different magnifications. Additionally, as a result of the nucleation and coalescence process, there is an occurrence of overgrown nanocrystallite spherical particles. These NPs have an average size of 110 nm. Interestingly, the particle size determined through TEM analysis exhibits a significant deviation when compared to the crystallite size obtained from the XRD analysis. This remarkable difference strongly suggests that the TiVO_4_/Co NPs are composed of self-assembled polycrystalline structures, indicating that the NPs possess a single-domain structure.^44,45^

Figure 4c displays a high-resolution TEM (HR-TEM) micrograph, revealing fringes with spacings of 0.323, corresponding to the (110) plane of TiVO_4_, indicating the highly crystalline nature of the film. Cobalt particles seem slightly agglomerated on the edge of the primary particle (yellow circles), which is common for samples prepared by the wet impregnation route.^46^ Furthermore, the corresponding SAED pattern in Figure 4d exhibits diffraction planes such as 110, 200, and 111, which correspond to the tetragonal crystal structure of TiVO_4_. These diffraction patterns confirm the polycrystalline nature of the prepared sample, whereas Figure 4e shows STEM images of Co-loaded TiVO_4_ with an overall uniform distribution of Co particles on the bare surface.

#### X-ray Photoelectron Spectroscopy Study

**Figure 5:**
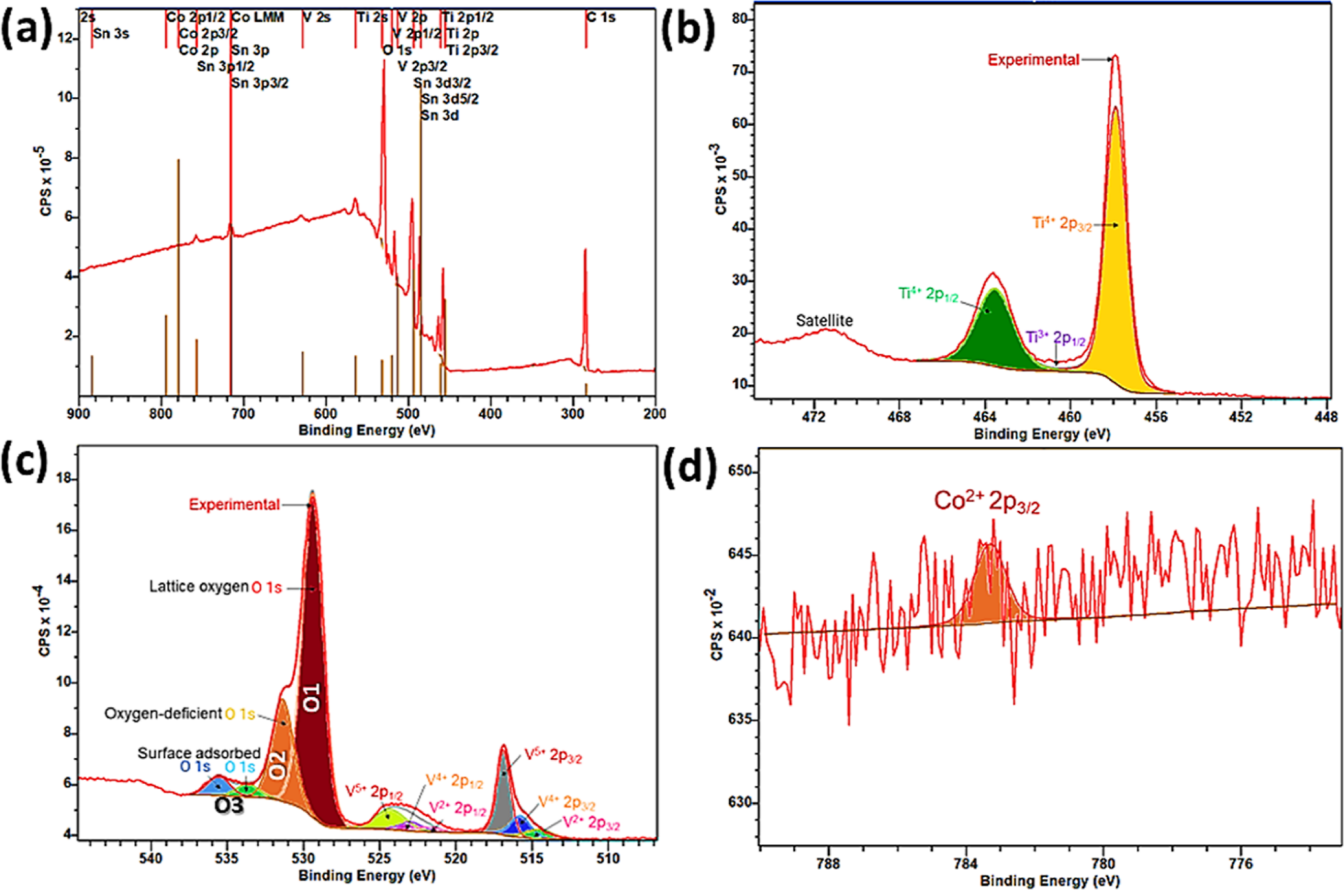
(a) XPS survey spectrum of the TiVO_4_/Co (3 mM) thin films on FTO glass and (b) XPS spectrum of spin–orbit deconvoluted peaks of Ti 2p, (c) V 2p with O 1s, and (d) Co 2p levels, respectively.

The surface composition of the Co-loaded TiVO_4_ sample was analyzed by XPS integrated peak area analysis, as shown in Figure 5. All binding energies were calibrated using the contaminant carbon (C 1s = 283.4 eV) as the reference. Figure 5a shows the surface spectrum of the sample, where Ti 2p, V 2p, O 1s, and Co 2p peaks were detected for the TiVO_4_/Co sample along with Sn 3d peaks, which is attributed to the FTO glass. Figure 5b displays characteristics of the Ti 2p spectrum of the TiVO_4_/Co sample composed of two dominating spin–orbit splitting of Ti 2p_1/2_ and Ti 2p_3/2_ peaks, located at 463.69 and 457.89, respectively, leading to a spin–orbit splitting energy of 5.8 eV that is characteristic of the +4 oxidation state of the Ti in the TiVO_4_ structure.^47^ Notably, the spin–orbit values are slightly higher than the typical Ti^4+^ spin–orbit values, probably due to the effect of the Co loading. In addition, a slight peak of Ti^3+^ was also noticed, originating probably due to the partial reduction of Ti^4+^ creating oxygen vacancies.

There is strong hybridization between V 2p and O 1s states and thus exposed various oxidation states of V 2p during TiVO_4_ formation, as shown in Figure 5c. V exhibits three significant oxidation states such as +5, +4, and +2, where the +5 states dominate.^48^ The binding energies at 514.63 and 521.70 eV correspond to V 2p_3/2_ and 2p_1/2_ for their +2 oxidation state, respectively. The V 2p_3/2_ and V 2p_1/2_ represent +4 oxidation states at 515.84 and 523.01 eV, respectively. The most stable and dominant V^5+^ oxidation states were found at 516.62 and 524.12 eV, corresponding to the spin–orbit binding energies of V^5+^ 2p_3/2_ and V^5+^ 2p_1/2_ states, respectively.^49^

On the other hand, Figure 5c also depicts the asymmetric O 1s core level spectrum for the Co-doped TiVO_4_ sample, which was deconvoluted into four peaks located at 529.37, 531.46, 533.72, and 535.44 eV, corresponding to lattice oxygen, oxygen vacancy, and chemisorbed or interstitial oxygen, respectively. The O 1 component on the lower binding energy side of the O 1s spectrum belongs to O^2–^ ions in Ti–O–V bonds of the tetragonal structure (lattice oxygen). The medium binding energy peak (O2) is associated with the O^2–^ ions in oxygen-deficient regions within the TiVO_4_ sample. Finally, the high binding energy portions (O3) are generally attributed to interstitial oxygen in TiVO_4_, which may include additional oxygen in the grain boundaries, such as chemisorbed or interstitial oxygen.^50^ Significantly, all these binding energy values of V and O are comparably higher than their standard form, probably due to the loading of Co^2+^, leading to the generation of an electronegativity difference, and thus, electron density around TiVO_4_ decreases and the binding energy increases.^51,52^

Moreover, Co^2+^ 2p states show an insignificant signal in its core-level spectrum analysis, as shown in Figure 5d. Because of the dilution effect, a weak and poorly resolved binding energy peak is observed at 783.29 eV, attributed to the Co^2+^ 2p_3/2_ state.^53^ However, Co^2+^ loading cannot be ensured while analyzing its spin–orbit spectrum, although the shifted binding energies of all other elemental oxidation states may indicate successful doping of Co^2+^ in TiVO_4_. Notably, the binding energies of Sn 3d_3/2_ and Sn 5d_5/2_ were assigned at 494.2 eV, and 485.8 eV was not shifted from their standard form due to Co^2+^ doping, representing the Sn^4+^ state originating from the FTO glass.^54^ Hence, through XPS analysis, effective loading of Co^2+^ in TiVO_4_ on FTO glass has been observed, and a moderate displacement of Ti and V metal binding energies indirectly supports this claim, while doping does not affect the substrate, FTO glass.

#### PEC Analysis of TiVO_4_/Co Thin Films

**Figure 6:**
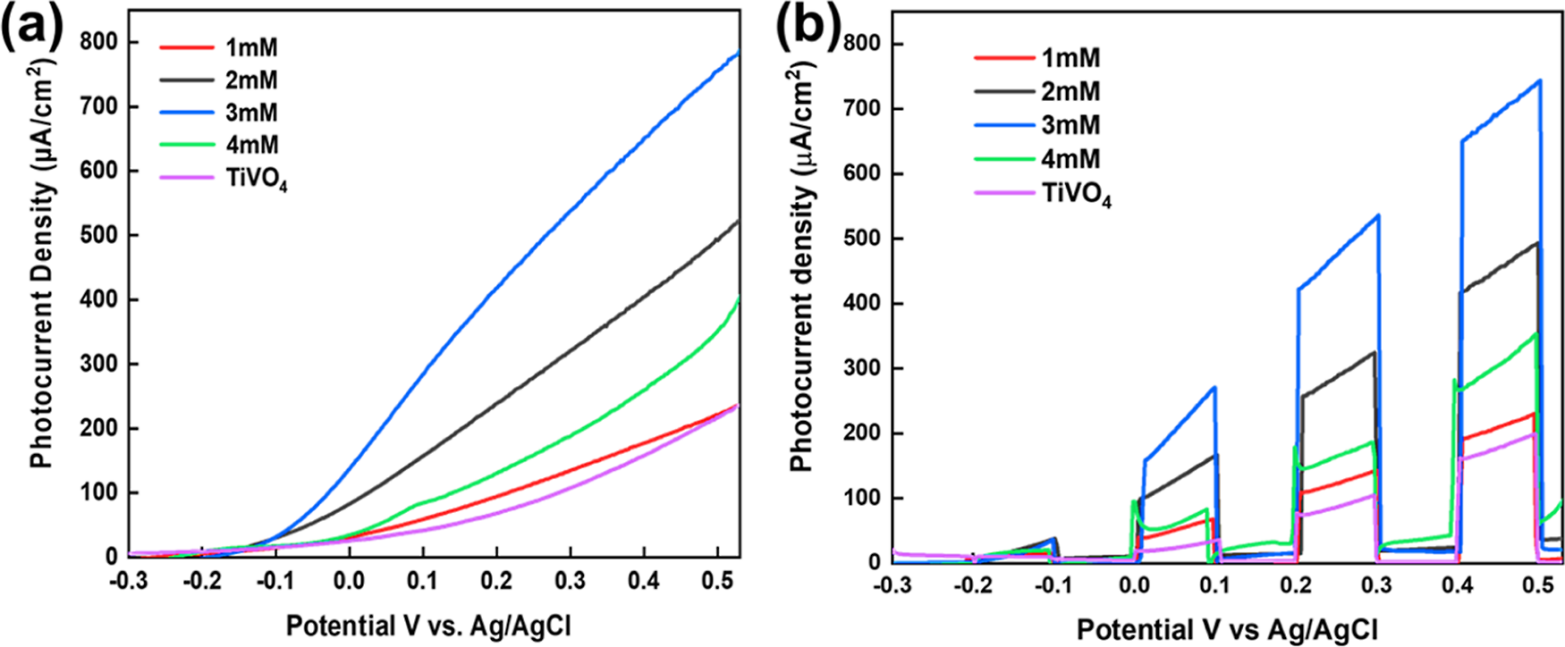
LSV plots of current density versus potential, referenced to Ag/AgCl, were obtained for TiVO_4_/Co films under two different conditions such as (a) continuous light illumination and (b) illumination with intermittent chopping, both conducted at an intensity of 100 mW/cm^2^, in a 1 M NaOH electrolyte with a pH of 13.6.

To understand the difference in PEC activities of various concentrations of Co loaded on TiVO_4_ electrodes, linear sweep voltammetry (LSV) was performed at a scan rate of 1 mV/s, and the results are recorded under light and chopped conditions in Figure 6a,6b, respectively. LSV plots revealed photocurrent (μA) vs potential (vs Ag/AgCl) trends of the untreated TiVO_4_ photoanode, which exhibited a photocurrent density of 80 μA/cm^2^ at 1.23 V vs RHE. Upon loading Co particles with concentrations of 1, 2, and 3 mM, gradual improvements in the photocurrent density reaching 120, 281, and 450 μA/cm^2^, respectively, were observed at 1.23 V vs RHE. This is due to the promotion of light absorption and increase of the active sites of the photocatalyst’s surface, as shown in absorption spectra.^55^ However, for the loading of a higher concentration of Co solution of 4 mM, a remarkable decrease in the photocurrent density value of 156 μA/cm^2^ was observed.^18,56^ The observed decrease in photocurrent density can be attributed to the higher loading effect of Co particles on the surface of the film, which leads to an inner filter effect. This effect blocks the active sites and reduces the interface between the photoanode and electrolyte, resulting in fast bulk recombination.

A comparative table of the impacts of the co-catalyst on the PEC performance of various photocatalysts is presented in Table 1. Based on our understanding of the loading effect of the co-catalyst on PEC performance, a Co concentration of 3 mM was determined to be the optimal concentration. At this concentration, the Co loading exhibited the maximum photocurrent, more than 5 times higher than that of bare TiVO_4_ and other composite electrodes. Therefore, further analyses were conducted to explore the exceptional performance observed at this concentration.

**Table 1:** Comparative Study of the Co NPs-Loaded TiVO_4_ Photoanode with the Latest Co-catalysts-Developed Thin Films for PEC Activity

serial no.	sample	particles deposition technique	photocurrent of the photocatalyst before depositing the co-catalyst (mA/cm^2^) (at1.23 V vs RHE)	photocurrent of the photocatalyst after depositing the co-catalyst (mA/cm^2^) (at 1.23 V vs RHE)	refs
1	Ag–LaFeO_3_	spin coating	0.0180	0.0380	(18)
2	Ni–LaFeO_3_	spin coating	0.0180	0.040	(19)
3	Co–BiVO_4_	electrochemical synthesis	0.313	0.460	(57)
4	Ag–ZnFe_2_O_4_	chemical water bath	0.100	0.240	(58)
5	Co–TiVO_4_	wet impregnation	0.080	0.450	this study

#### EIS Analysis

**Figure 7:**
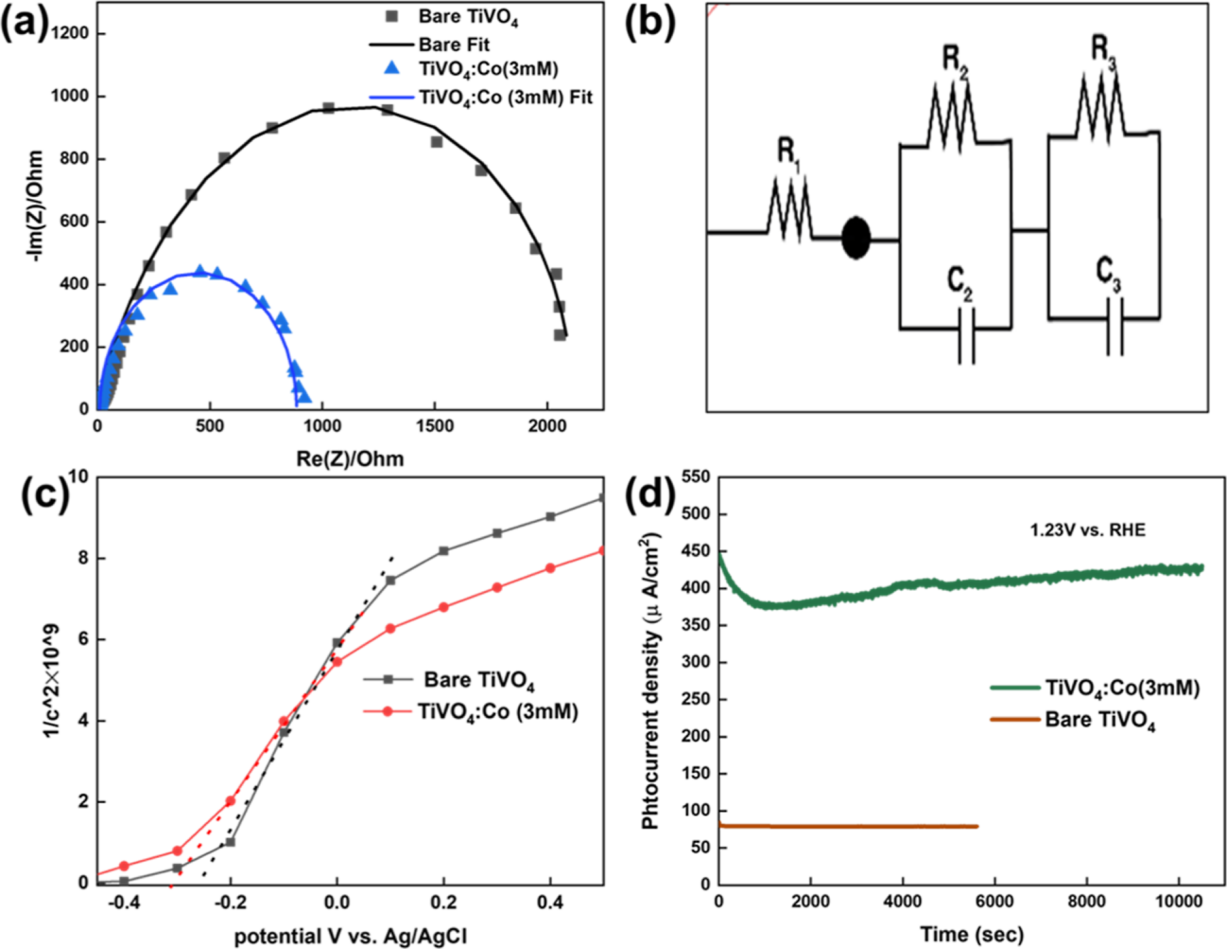
(a) Nyquist plots, (b) corresponding equivalent circuit, (c) Mott–Schottky plots and (d) photocurrent stability plots of prepared TiVO_4_ and TiVO_4_/Co (3 mM) thin films.

EIS is a robust measurement for investigating the interfacial properties of the interface between electrodes and electrolytes and is employed in most energy applications.^59^Figure 7a displays the fitting and experimental Nyquist plots of bare TiVO_4_ and Co loaded onto TiVO_4_ films under 1 SUN illumination (100 mW/cm^2^). Both are similar, consisting of semicircles with an overlap between fitting and experiment plots.^60^ To compare the resistances values of the two photoanodes, the same equivalent circuit (*R*_1_ + *R*_2_/C_2_ + R_3_/C_3_) was used, as shown in Figure 7b. TiVO_4_/Co (3 mM) has a smaller semi-circular radius, producing the highest photocatalytic performance. The values of *R*_1_ and *R*_2_ for TiVO_4_/Co (3 mM) are 11.54 and 854.3 Ω, respectively, which are lower than were found for bare TiVO_4_ (*R*_1_ = 12.41 Ω, *R*_2_ = 1983 Ω), indicating to a significant reduction of transfer charge resistance, better charge transport at the electrode/electrolyte interface, and faster surface reaction kinetics, caused by the Co NPs.^61^

To further investigate the enhancements in prepared TiVO_4_/Co (3 mM), a Mott–Schottky plot was obtained to determine the space charge capacitance and flat band potential (V_b_), as depicted in Figure 7c. Previous research estimated the flat band potential of bare TiVO_4_ film to be −0.26 V vs Ag/AgCl, while that of TiVO_4_/Co (3 mM) was determined to be −0.30 V vs Ag/AgCl. This negative shift in the flat band potential indicates the formation of a small barrier in TiVO_4_/Co (3 mM) heterostructures, which is consistent with observations from SEM images. Interestingly, the Co catalyst acts as an electron reservoir on the surface of the photocatalyst by establishing an inherent barrier layer that prevents direct electron transfer from the TiVO_4_ conduction band to the electrolyte, thereby suppressing charge recombination at the interface. However, a higher loading amount of Co particles can allow them to act as recombination centers, leading to agglomeration and formation of a thicker barrier layer, hindering charge transfer, and resulting in a lower photocurrent density, as observed in SEM images.

The carrier density of the prepared TiVO_4_/Co was calculated to be 9 × 10^20^ cm^–3^, which is higher than that of bare TiVO_4_ (7.7 × 10^20^ cm^–3^). This higher carrier density suggests accelerated charge transfer, thus improving the PEC performance. Figure 7d demonstrates the stability of TiVO_4_/Co (3 mM) under 3 h of illumination at a fixed applied bias potential of 1.23 V vs RHE. The TiVO_4_/Co (3 mM) film exhibits significantly longer stability than the bare film, which is approximately twice as long. However, during the initial 20 min under illumination, a minor and insignificant decrease in PEC efficiency is observed.

#### Photocatalytic Analysis of TiVO_4_/Co Thin Films

The photocatalytic activity of metal oxide semiconductors is influenced by several factors, including surface area, which is inversely proportional to size and directly related to the band gap energy of the semiconductor, surface morphology (specifically surface roughness), defect concentration, dopant content, and electrochemical properties.^62^ In this study, the TiVO_4_/Co (3 mM) film exhibited the highest photocurrent, making it the chosen candidate for testing its effectiveness in degrading dyes through photocatalysis.

**Figure 8:**
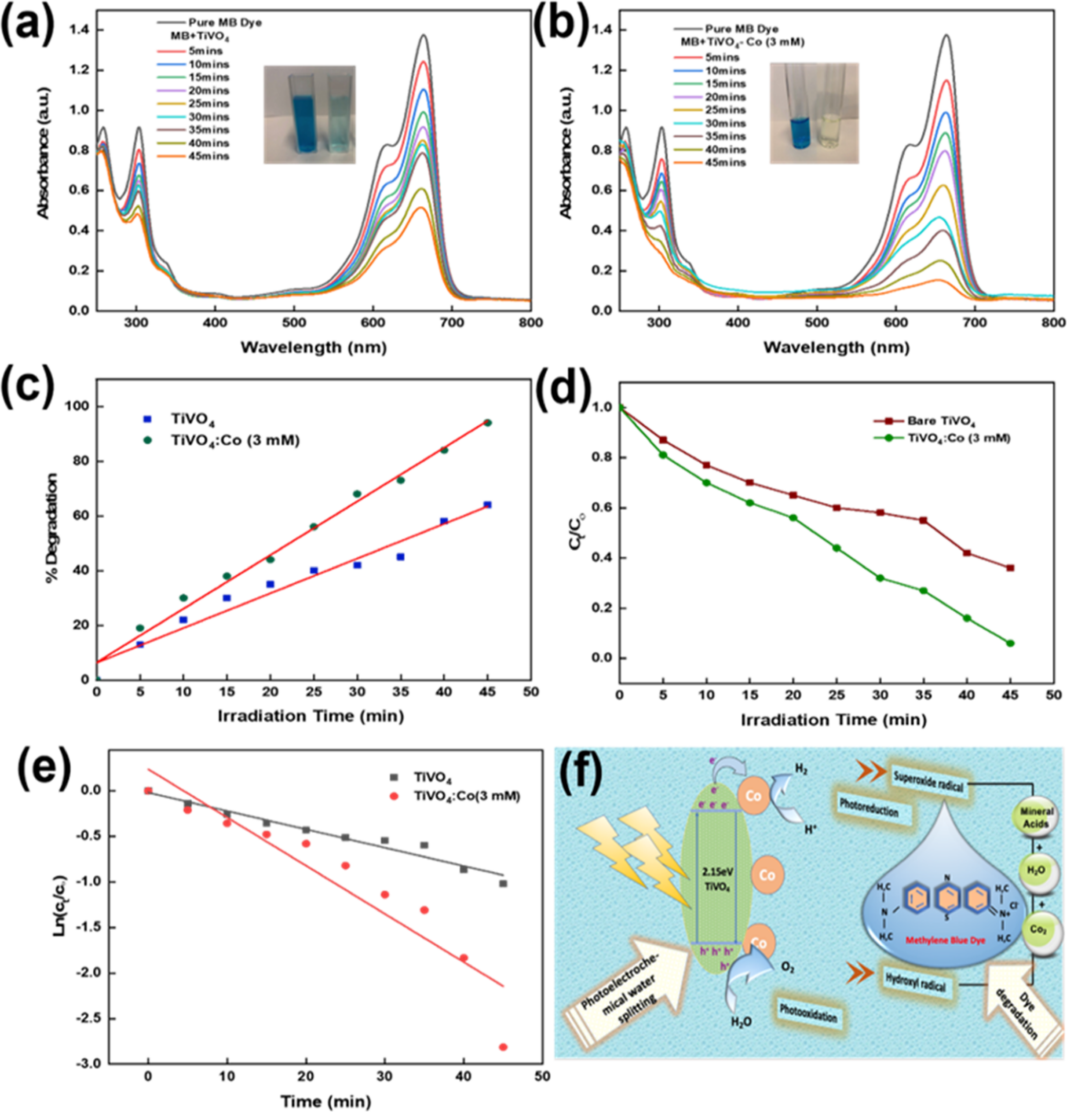
Absorption spectra of MB measured after exposure to light for varying durations using (a) TiVO_4_ and (b) TiVO_4_/Co (3 mM), respectively, (c) percentage degradation of MB calculated for TiVO_4_ and TiVO_4_/Co (3 mM), (d) corresponding plot of comparative photocatalytic MB degradation rate, (e) pseudo-first-order kinetic linear plot for prepared TiVO_4_ and TiVO_4_/Co (3 mM), and (f) schematic representation of the photocatalytic mechanism under visible light irradiation.

The high efficiency of any photocatalytic dye degradation system is governed by sufficient light harvesting, photo-generated charge carriers, and charge transfer, which are relatively slow in some photocatalysts. For this purpose, we used MB as the model reaction due to its chemical stability and contamination of wastewater. Herein, co-catalysts of bare and modified films were investigated for their photocatalytic activity on removing 10 mg/L MB by degradation under illumination. Figure 8a,b displays the kinetics of the dye degradation of MB using TiVO_4_ and TiVO_4_/Co (3 mM) as catalysts, respectively. The spectra indicate that the presence of the Co particles significantly enhanced the decrease of maximum absorbance intensity recorded at 665 nm of MB bands with a tested time faster than was observed in the TiVO_4_ film until it degraded completely after 45 min. The inserted images display MB dyes in their pure blue color before and after being illuminated, as well as their appearance after being exposed to light for 45 min. The reduction in MB degradation was 97% for the bare system and 60% for the Co-loaded bare system, as shown in Figure 8c. Figure 8d illustrates the comparable rates of photodegradation for TiVO_4_/Co in comparison to that of bare TiVO_4_ photocathodes. The results indicate that TiVO_4_/Co exhibits a significantly faster and steeper degradation rate throughout the illumination period when compared to bare TiVO_4_. The disparity in performance between the two cases can be attributed to the presence of Co. The inclusion of Co^2+^ on the surface facilitates the rapid degradation of MB by enhancing charge separation and transfer at the interface, as indicated by the EIS plots. In contrast, MB degradation is slowed in TiVO_4_ due to the quick recombination of charges. Figure 8e demonstrates linear regressions of pseudo-first-order kinetics for different photocatalysis systems. The calculated reaction rate constants increased from 0.019 to 0.044 min^–1^ after loading Co NPs onto the bare surface. This suggests that Co^2+^ can generate additional free radicals such as OH^•^, resulting in an overall improvement in photocatalytic activation.

Figure 8f illustrates the photocatalytic activity mechanism of Co-loaded TiVO_4_. Despite their similar band gap energies (2.18–2.15 eV), TiVO_4_/Co exhibits slightly more negative conduction band (CB) potentials than TiVO_4_. By loading Co particles onto the surface of the photocatalyst, excited electrons are transferred from the TiVO_4_ conduction band to the Co particles on the surface. This transfer prevents recombination within the bulk film. Conversely, photo-generated holes in the valence band remain on the photocatalyst. Consequently, the accumulated electrons on the Co particles participate in reduction reactions, while the holes diffuse to the photocatalyst’s surface and contribute to oxidation reactions, driving overall water splitting reactions. In the dye degradation system, the photo-generated holes react with water molecules to form hydroxyl radicals (OH^•^), which react with dye molecules, generating carbon-centered radicals. These radicals efficiently convert to CO_2_ upon interacting with oxygen molecules. Furthermore, the accumulated electrons on the Co particles produce superoxide radicals (O_2_^–^), which also react with oxygen molecules, generating more reactive oxygen species and facilitating the degradation of MB dye molecules. Notably, during our stability testing, the prepared film exhibited reduced photocatalytic efficiency over 3 h, primarily due to light effects. This decrease in efficiency can be attributed to the limitations of the deposition technique employed. While the spray pyrolysis method effectively produces satisfactory films, it falls short in terms of long-term stability.

## Conclusions

Introducing Co NPs into TiVO_4_ photoanodes resulted in enhanced performance and absorption spectra, which depended on the quantity of Co utilized. Incorporating Co^2+^ ions in the solution further improved the TiVO_4_ photoanode by reducing recombination rates and facilitating electron transfers. The concentration of Co loading had a noteworthy impact on the performance of the photocatalysts, with the highest performance observed at a Co loading of 3 mM. The addition of TiVO_4_/Co also slightly decreased the band gap of the bare film, as indicated by a negative shift in the potential scan rate on the Mott–Schottky plot. The improved PEC activity of the TiVO_4_ photoanode with Co (3 mM) incorporation was attributed to the abundance of oxygen vacancies and improved alignment of primary TiVO_4_ particles. However, beyond the optimal loading amount of 4 mM, the photocurrent density decreased significantly due to cobalt agglomeration and accelerated recombination of charge carriers. Furthermore, the cost-effective and straightforward method of incorporating co-catalyst NPs provided strong evidence supporting the application of photoanodes, making them a promising choice for other photocatalysts. Compared to more durable deposition methods such as physical vapor deposition (PVD) and electrodeposition, the current film deposition technique necessitates further modifications, particularly when considering long-term applications. Hence, our future goal is to utilize PVD techniques to develop thin films that offer improved reusability and long-term stability for photoelectrodes. The use of physical deposition techniques requires additional modifications to ensure prolonged usage.

## References

[ref1] Wang Y.; Zhang J.; Balogun M.-S.; Tong Y.; Huang Y. Oxygen vacancy–based metal oxides photoanodes in photoelectrochemical water splitting. Mater. Today Sustain. 2022, 18, 100118 10.1016/j.mtsust.2022.100118.

[ref2] Shi H.; Guo H.; Wang S.; Zhang G.; Hu Y.; Jiang W.; Liu G. Visible Light Photoanode Material for Photoelectrochemical Water Splitting: A Review of Bismuth Vanadate. Energy Fuels 2022, 36, 11404–11427. 10.1021/acs.energyfuels.2c00994.

[ref3] Li S.; Xu W.; Meng L.; Tian W.; Li L. Recent Progress on Semiconductor Heterojunction-Based Photoanodes for Photoelectrochemical Water Splitting. Small Sci. 2022, 2, 2100112 10.1002/smsc.202100112. PMC11935891 40212603

[ref4] Fareza A. R.; Nugroho F. A. A.; Abdi F.; Fauzia V. Nanoscale metal oxides–2D materials heterostructures for photoelectrochemical water splitting—a review. J. Mater. Chem. A 2022, 10, 8656–8686. 10.1039/d1ta10203f.

[ref5] Fujishima A.; Honda K. Electrochemical photolysis of water at a semiconductor electrode. Nature 1972, 238, 37–38. 10.1038/238037a0. 12635268

[ref6] Bae D.; Seger B.; Vesborg P. C.; Hansen O.; Chorkendorff I. Strategies for stable water splitting via protected photoelectrodes. Chem. Soc. Rev. 2017, 46, 1933–1954. 10.1039/c6cs00918b. 28246670

[ref7] Tahir A. A.; Wijayantha K. G. U.; Saremi-Yarahmadi S.; Mazhar M.; McKee V. Nanostructured α-Fe_2_O_3_ Thin Films for Photoelectrochemical Hydrogen Generation. Chem. Mater. 2009, 21, 3763–3772. 10.1021/cm803510v.

[ref8] Hong T.; Liu Z.; Zheng X.; Zhang J.; Yan L. Efficient photoelectrochemical water splitting over Co3O4 and Co3O4/Ag composite structure. Appl. Catal., B 2017, 202, 454–459. 10.1016/j.apcatb.2016.09.053.

[ref9] Hoang S.; Berglund S. P.; Hahn N. T.; Bard A. J.; Mullins C. B. Enhancing visible light photo-oxidation of water with TiO2 nanowire arrays via cotreatment with H2 and NH3: synergistic effects between Ti3+ and N. J. Am. Chem. Soc. 2012, 134, 3659–3662. 10.1021/ja211369s. 22316385

[ref10] Hernández S.; Thalluri S. M.; Sacco A.; Bensaid S.; Saracco G.; Russo N. Photo-catalytic activity of BiVO4 thin-film electrodes for solar-driven water splitting. Appl. Catal., A 2015, 504, 266–271. 10.1016/j.apcata.2015.01.019.

[ref11] Mandal H.; Shyamal S.; Hajra P.; Bera A.; Sariket D.; Kundu S.; Bhattacharya C. Development of ternary iron vanadium oxide semiconductors for applications in photoelectrochemical water oxidation. RSC Adv. 2016, 6, 4992–4999. 10.1039/c5ra22586h.

[ref12] Kalanur S. S.; Seo H. Facile growth of compositionally tuned copper vanadate nanostructured thin films for efficient photoelectrochemical water splitting. Appl. Catal., B 2019, 249, 235–245. 10.1016/j.apcatb.2019.02.069.

[ref13] Chen H. M.; Chen C. K.; Chang Y. C.; Tsai C. W.; Liu R. S.; Hu S. F.; Chang W. S.; Chen K. H. Quantum dot monolayer sensitized ZnO nanowire-array photoelectrodes: true efficiency for water splitting. Angew. Chem. 2010, 122, 6102 10.1002/ange.201001827. 20632423

[ref14] Chen D.; Liu Z. Dual-axial gradient doping (Zr and Sn) on hematite for promoting charge separation in photoelectrochemical water splitting. ChemSusChem 2018, 11, 3438–3448. 10.1002/cssc.201801614. 30098118

[ref15] Dong Z.; Ding D.; Li T.; Ning C. Ni-doped TiO2 nanotubes photoanode for enhanced photoelectrochemical water splitting. Appl. Surf. Sci. 2018, 443, 321–328. 10.1016/j.apsusc.2018.03.031.

[ref16] Ding Q.; Gou L.; Wei D.; Xu D.; Fan W.; Shi W. Metal-organic framework derived Co3O4/TiO2 heterostructure nanoarrays for promote photoelectrochemical water splitting. Int. J. Hydrogen Energy 2021, 46, 24965–24976. 10.1016/j.ijhydene.2021.05.065.

[ref17] Alhabradi M.; Nundy S.; Ghosh A.; Tahir A. A. Vertically Aligned CdO-Decked α-Fe2O3 Nanorod Arrays by a Radio Frequency Sputtering Method for Enhanced Photocatalytic Applications. ACS Omega 2022, 7, 28396 10.1021/acsomega.2c02996. 35990474 PMC9386802

[ref18] Pawar G. S.; Elikkottil A.; Seetha S.; Reddy K. S.; Pesala B.; Tahir A. A.; Mallick T. K. Enhanced photoactivity and hydrogen generation of LaFeO3 photocathode by plasmonic silver nanoparticle incorporation. ACS Appl. Energy Mater. 2018, 1, 3449–3456. 10.1021/acsaem.8b00628.

[ref19] Pawar G. S.; Elikkottil A.; Pesala B.; Tahir A. A.; Mallick T. K. Plasmonic nickel nanoparticles decorated on to LaFeO3 photocathode for enhanced solar hydrogen generation. Int. J. Hydrogen Energy 2019, 44, 578–586. 10.1016/j.ijhydene.2018.10.240.

[ref20] Wang C.-C.; Chou P.-H.; Yu Y.-H.; Kei C.-C. Deposition of Ni nanoparticles on black TiO2 nanowire arrays for photoelectrochemical water splitting by atomic layer deposition. Electrochim. Acta 2018, 284, 211–219. 10.1016/j.electacta.2018.07.164.

[ref21] Ran J.; Zhang J.; Yu J.; Jaroniec M.; Qiao S. Z. Earth-abundant cocatalysts for semiconductor-based photocatalytic water splitting. Chem. Soc. Rev. 2014, 43, 7787–7812. 10.1039/c3cs60425j. 24429542

[ref22] Jiang W.; Bai S.; Wang L.; Wang X.; Yang L.; Li Y.; Liu D.; Wang X.; Li Z.; Jiang J.; et al. Integration of multiple plasmonic and co-catalyst nanostructures on TiO2 nanosheets for visible-near-infrared photocatalytic hydrogen evolution. Small 2016, 12, 1640–1648. 10.1002/smll.201503552. 26833931

[ref23] Hong D.; Cao G.; Zhang X.; Qu J.; Deng Y.; Liang H.; Tang J. Construction of a Pt-modified chestnut-shell-like ZnO photocatalyst for high-efficiency photochemical water splitting. Electrochim. Acta 2018, 283, 959–969. 10.1016/j.electacta.2018.05.051.

[ref24] Ma D.; Shi J.-W.; Sun D.; Zou Y.; Cheng L.; He C.; Wang H.; Niu C.; Wang L. Au decorated hollow ZnO@ ZnS heterostructure for enhanced photocatalytic hydrogen evolution: The insight into the roles of hollow channel and Au nanoparticles. Appl. Catal., B 2019, 244, 748–757. 10.1016/j.apcatb.2018.12.016.

[ref25] Reddy N. L.; Kumar S.; Krishnan V.; Sathish M.; Shankar M. Multifunctional Cu/Ag quantum dots on TiO2 nanotubes as highly efficient photocatalysts for enhanced solar hydrogen evolution. J. Catal. 2017, 350, 226–239. 10.1016/j.jcat.2017.02.032.

[ref26] Hu X.; Li Y.; Wei X.; Wang L.; She H.; Huang J.; Wang Q. Preparation of double-layered Co– Ci/NiFeOOH co-catalyst for highly meliorated PEC performance in water splitting. Advanced Powder Materials 2022, 1, 100024 10.1016/j.apmate.2021.11.010.

[ref27] Moakhar R. S.; Kushwaha A.; Jalali M.; Goh G. K. L.; Dolati A.; Ghorbani M. Enhancement in solar driven water splitting by Au–Pd nanoparticle decoration of electrochemically grown ZnO nanorods. J. Appl. Electrochem. 2016, 46, 819–827. 10.1007/s10800-016-0981-x.

[ref28] Reichert R.; Jusys Z.; Behm R. Jr Au/TiO2 photo (electro) catalysis: the role of the Au cocatalyst in photoelectrochemical water splitting and photocatalytic H2 evolution. J. Phys. Chem. C 2015, 119, 24750–24759. 10.1021/acs.jpcc.5b08428.

[ref29] Siavash Moakhar R.; Jalali M.; Kushwaha A.; Kia Liang Goh G.; Riahi-Noori N.; Dolati A.; Ghorbani M. AuPd bimetallic nanoparticle decorated TiO2 rutile nanorod arrays for enhanced photoelectrochemical water splitting. J. Appl. Electrochem. 2018, 48, 995–1007. 10.1007/s10800-018-1231-1.

[ref30] Anjugam Vandarkuzhali S. A.; Pugazhenthiran N.; Mangalaraja R.; Sathishkumar P.; Viswanathan B.; Anandan S. Ultrasmall plasmonic nanoparticles decorated hierarchical mesoporous TiO2 as an efficient photocatalyst for photocatalytic degradation of textile dyes. ACS Omega 2018, 3, 9834–9845. 10.1021/acsomega.8b01322. 31459112 PMC6644734

[ref31] Marimuthu S.; Antonisamy A. J.; Malayandi S.; Rajendran K.; Tsai P.-C.; Pugazhendhi A.; Ponnusamy V. K. Silver nanoparticles in dye effluent treatment: A review on synthesis, treatment methods, mechanisms, photocatalytic degradation, toxic effects and mitigation of toxicity. J. Photochem. Photobiol., B 2020, 205, 111823 10.1016/j.jphotobiol.2020.111823. 32120184

[ref32] Thao L. T.; Nguyen T. V.; Nguyen V. Q.; Phan N. M.; Kim K. J.; Huy N. N.; Dung N. T. Orange G degradation by heterogeneous peroxymonosulfate activation based on magnetic MnFe2O4/α-MnO2 hybrid. J. Environ. Sci. 2023, 124, 379–396. 10.1016/j.jes.2021.10.008. 36182147

[ref33] Wang J.; Osterloh F. E. Limiting factors for photochemical charge separation in BiVO 4/Co 3 O 4, a highly active photocatalyst for water oxidation in sunlight. J. Mater. Chem. A 2014, 2, 9405–9411. 10.1039/c4ta01654h.

[ref34] Wang X.; Zhang S.; Peng B.; Wang H.; Yu H.; Peng F. Enhancing the photocatalytic efficiency of TiO2 nanotube arrays for H2 production by using non-noble metal cobalt as co-catalyst. Mater. Lett. 2016, 165, 37–40. 10.1016/j.matlet.2015.11.103.

[ref35] Khan H. R.; Aamir M.; Malik M. A.; Tahir A. A.; Akram B.; Murtaza G.; Choudhary M. A.; Akhtar J. Chemically vaporized cobalt incorporated wurtzite as photoanodes for efficient photoelectrochemical water splitting. Mater. Sci. Semicond. Process. 2019, 101, 223–229. 10.1016/j.mssp.2019.06.002.

[ref36] Alruwaili M.; Roy A.; Nundy S.; Tahir A. A. Fabrication of TiVO 4 photoelectrode for photoelectrochemical application. RSC Adv. 2022, 12, 34640–34651. 10.1039/d2ra05894d. 36545617 PMC9717350

[ref37] Lin H.-Y.; Yang H.-C.; Wang W.-L. Synthesis of mesoporous Nb2O5 photocatalysts with Pt, Au, Cu and NiO cocatalyst for water splitting. Catal. Today 2011, 174, 106–113. 10.1016/j.cattod.2011.01.052.

[ref38] Han X.; Xu D.; An L.; Hou C.; Li Y.; Zhang Q.; Wang H. Ni-Mo nanoparticles as co-catalyst for drastically enhanced photocatalytic hydrogen production activity over g-C3N4. Appl. Catal., B 2019, 243, 136–144. 10.1016/j.apcatb.2018.10.003.

[ref39] Ayala P.; Giesriegl A.; Nandan S. P.; Myakala S. N.; Wobrauschek P.; Cherevan A. Isolation strategy towards earth-abundant single-site co-catalysts for photocatalytic hydrogen evolution reaction. Catalysts 2021, 11, 417 10.3390/catal11040417.

[ref40] Zhong D. K.; Cornuz M.; Sivula K.; Grätzel M.; Gamelin D. R. Photo-assisted electrodeposition of cobalt–phosphate (Co–Pi) catalyst on hematite photoanodes for solar water oxidation. Energy Environ. Sci. 2011, 4, 1759–1764. 10.1039/c1ee01034d.

[ref41] Ma B.; Li X.; Li D.; Lin K. A difunctional photocatalytic H2 evolution composite co-catalyst tailored by integration with earth-abundant material and ultralow amount of noble metal. Appl. Catal., B 2019, 256, 117865 10.1016/j.apcatb.2019.117865.

[ref42] Touqeer M.; Baig M. M.; Aadil M.; Agboola P. O.; Shakir I.; Aboud M. F. A.; Warsi M. F. New Co-MnO based Nanocrsytallite for photocatalysis studies driven by visible light. J. Taibah Univ. Sci. 2020, 14, 1580–1589. 10.1080/16583655.2020.1846966.

[ref43] He H.; Liao A.; Guo W.; Luo W.; Zhou Y.; Zou Z. State-of-the-art progress in the use of ternary metal oxides as photoelectrode materials for water splitting and organic synthesis. Nano Today 2019, 28, 100763 10.1016/j.nantod.2019.100763.

[ref44] Li Q.; Kartikowati C. W.; Horie S.; Ogi T.; Iwaki T.; Okuyama K. Correlation between particle size/domain structure and magnetic properties of highly crystalline Fe3O4 nanoparticles. Sci. Rep. 2017, 7, 9894 10.1038/s41598-017-09897-5. 28855564 PMC5577113

[ref45] Babu M. M. H.; Podder J.; Tofa R. R.; Ali L. Effect of Co doping in tailoring the crystallite size, surface morphology and optical band gap of CuO thin films prepared via thermal spray pyrolysis. Surf. Interfaces 2021, 25, 101269 10.1016/j.surfin.2021.101269.

[ref46] Maeda K.; Sakamoto N.; Ikeda T.; Ohtsuka H.; Xiong A.; Lu D.; Kanehara M.; Teranishi T.; Domen K. Preparation of Core–Shell-Structured Nanoparticles (with a Noble-Metal or Metal Oxide Core and a Chromia Shell) and Their Application in Water Splitting by Means of Visible Light. Chem.—Eur. J. 2010, 16, 7750–7759. 10.1002/chem.201000616. 20564294

[ref47] Krishnapriya R.; Nizamudeen C.; Saini B.; Mozumder M. S.; Sharma R. K.; Mourad A.-H. MOF-derived Co2+-doped TiO2 nanoparticles as photoanodes for dye-sensitized solar cells. Sci. Rep. 2021, 11, 16265 10.1038/s41598-021-95844-4. 34381114 PMC8358052

[ref48] Yu D.; Wei W.; Wei M.; Wang F.; Liang X.; Sun S.; Gao M.; Zhu Q. Research on the electrochromic properties of Mxene intercalated vanadium pentoxide xerogel films. J. Solid State Electrochem. 2022, 26, 1399–1407. 10.1007/s10008-022-05171-5.

[ref49] Silversmit G.; Depla D.; Poelman H.; Marin G. B.; De Gryse R. Determination of the V2p XPS binding energies for different vanadium oxidation states (V5+ to V0+). J. Electron Spectrosc. Relat. Phenom. 2004, 135, 167–175. 10.1016/j.elspec.2004.03.004.

[ref50] Nag P.; Das P. P.; Roy A.; Devi P. S. Iron antimonate quantum dots exhibiting tunable visible light emission. New J. Chem. 2017, 41, 1436–1446. 10.1039/c6nj02767a.

[ref51] Cao S.; Zhang X.; Komesu T.; Chen G.; Schmid A. K.; Yue L.; Tanabe I.; Echtenkamp W.; Wang Y.; Binek C.; et al. Low temperature growth of cobalt on Cr2O3 (0 0 0 1). J. Phys.: Condens. Matter 2016, 28, 046002 10.1088/0953-8984/28/4/046002. 26732426

[ref52] Jia H.; Chen C.; Oladele O.; Tang Y.; Li G.; Zhang X.; Yan F. Cobalt doping of tin disulfide/reduced graphene oxide nanocomposites for enhanced pseudocapacitive sodium-ion storage. Commun. Chem. 2018, 1, 86 10.1038/s42004-018-0086-z.

[ref53] Charron G.; Giusti A.; Mazerat S.; Mialane P.; Gloter A.; Miserque F.; Keita B.; Nadjo L.; Filoramo A.; Riviere E.; et al. Assembly of a magnetic polyoxometalate on SWNTs. Nanoscale 2010, 2, 139–144. 10.1039/b9nr00190e. 20648376

[ref54] Indira Gandhi T.; Ramesh Babu R.; Ramamurthi K.; Arivanandhan M. Electrical and optical properties of Co 2+: SnO 2 thin films deposited by spray pyrolysis technique. J. Mater. Sci.: Mater. Electron. 2016, 27, 1662–1669. 10.1007/s10854-015-3938-7.

[ref55] Tran P. D.; Xi L.; Batabyal S. K.; Wong L. H.; Barber J.; Chye Loo J. S. Enhancing the photocatalytic efficiency of TiO 2 nanopowders for H 2 production by using non-noble transition metal co-catalysts. Phys. Chem. Chem. Phys. 2012, 14, 11596–11599. 10.1039/c2cp41450c. 22828930

[ref56] Wang P.; Sheng Y.; Wang F.; Yu H. Synergistic effect of electron-transfer mediator and interfacial catalytic active-site for the enhanced H2-evolution performance: A case study of CdS-Au photocatalyst. Appl. Catal., B 2018, 220, 561–569. 10.1016/j.apcatb.2017.08.080.

[ref57] Miao Y.; Liu J.; Chen L.; Sun H.; Zhang R.; Guo J.; Shao M. Single-atomic-Co cocatalyst on (040) facet of BiVO4 toward efficient photoelectrochemical water splitting. Chem. Eng. J. 2022, 427, 131011 10.1016/j.cej.2021.131011.

[ref58] Lan Y.; Liu Z.; Liu G.; Guo Z.; Ruan M.; Rong H.; Li X. 1D ZnFe2O4 nanorods coupled with plasmonic Ag, Ag2S nanoparticles and Co-Pi cocatalysts for efficient photoelectrochemical water splitting. Int. J. Hydrogen Energy 2019, 44, 19841–19854. 10.1016/j.ijhydene.2019.05.184.

[ref59] To N. V.; Nguyen K. V.; Nguyen H. S.; Luong S. T.; Doan P. T.; Nguyen T. H. T.; Ngo Q. Q.; Nguyen N. V. P2-type layered structure Na1. 0Li0. 2Mn0. 7Ti0. 1O2 as a superb electrochemical performance cathode material for sodium-ion batteries. J. Electroanal. Chem. 2021, 880, 114834 10.1016/j.jelechem.2020.114834.

[ref60] Quyen N. Q.; Van Nguyen T.; Thang H. H.; Thao P. M.; Van Nghia N. Carbon coated NaLi0. 2Mn0. 8O2 as a superb cathode material for sodium ion batteries. J. Alloys Compd. 2021, 866, 158950 10.1016/j.jallcom.2021.158950.

[ref61] Lu X.; Xiao J.; Peng L.; Zhang L.; Zhan G. Enhancement in the photoelectrochemical performance of BiVO4 photoanode with high (040) facet exposure. J. Colloid Interface Sci. 2022, 628, 726–735. 10.1016/j.jcis.2022.07.189. 35944303

[ref62] Dung N. T.; Thu T. V.; Van Nguyen T.; Thuy B. M.; Hatsukano M.; Higashimine K.; Maenosono S.; Zhong Z. Catalytic activation of peroxymonosulfate with manganese cobaltite nanoparticles for the degradation of organic dyes. RSC Adv. 2020, 10, 3775–3788. 10.1039/c9ra10169a. 35492672 PMC9048426

